# Supplementary material for: Study on the Role of Cytc in Response to BmNPV Infection in Silkworm, Bombyx mori (Lepidoptera)
Source: Int J Mol Sci. 2019 Sep 4;20(18):4325. doi: 10.3390/ijms20184325 (PMC6770455; doi:10.3390/ijms20184325)
Supplement: Supplementary file 1 [file ijms-20-04325-s001.pdf]

International Journal of
Molecular Sciences

MDPI

Supplementary materials

```
     cccgggctgcaggatcgaattcgcaacgcgacgattcacgttgtctcgattaagtgtctaa

     aatttggtgattttggcaaacctaaaaaatttggctgttacattaattataaaaatttaaa

1    ATGGGTGTACCTGCAGGAAACGCTGAAAATGGAAAGAAAATTTTTGTACAACGATGTGCC
1     M  G  V  P  A  G  N  A  E  N  G  K  K  I  F  V  Q  R  C  A

61   CAGTGCCACACTGTTGAAGCTGGTGGCAAACACAAAGTAGGACAGAATCTACATGGATTC
21    Q  C  H  T  V  E  A  G  G  K  H  K  V  G  Q  N  L  H  G  F

121  TTTGGCCGAAAAACTGGCCAGGCTGCAGGATTCTCATACTCCGATGCCAATAAAGCTAAG
41    F  G  R  K  T  G  Q  A  A  G  F  S  Y  S  D  A  N  K  A  K

181  GGCATTACATGGAATGACGACACTCTCTTTGAATATCTTGAGAATCCCAAGAAATACATC
61    G  I  T  W  N  D  D  T  L  F  E  Y  L  E  N  P  K  K  Y  I

241  CCTGGAACCAAGATGGTGTTTGCTGGACTCAAGAAGGCAAATGAGCGTGCTGACCTTATT
81    P  G  T  K  M  V  F  A  G  L  K  K  A  N  E  R  A  D  L  I

301  GCCTATCTCAAATCTGCTACCAAGTAAttttaaagtaatagaaaattccataagaagcat
101   A  Y  L  K  S  A  T  K  *

     tttccataacatttctggaatttacttcttatttagcccaatcagtatgatcagttatgt

     attcagcaatgttacaattgcactataatattaaagtatttgttagttccaaatcacaac

     acaatc
```

Figure S1. The full-length nucleotide sequence and deduced amino acid sequence of *Bmcytc*. The start and termination codons are denoted in the black frame. The functional domain of the Bmcytc protein is underlined.

```
Bombyx mori                  MGVPAGNAENGKKIFVQRCAQCHTVEAGGKHKVGPNLHGFFGRKTGQAAGFSYSD      55
Amyelois transitella         MGIPAGNAENGKKIFVQRCAQCHTVEAGGKHKVGPNLNGFFGRKTGQAAGFSYSD      55
Bactrocera dorsalis          MGVPAGDVEKGKKLFVQRCAQCHTVEAGGKHKVGPNLHGLFGRKTGQAAGFSYTD      55
Danaus plexippus plexippus   MGVPAGNAENGKKIFVQRCAQCHTVEAGGKHKVGPNLHGFFGRKTGQASGFNYSE      55
Hyposmocoma kahamanoa        MGVPAGNAENGKKIFVQRCAQCHTVEAGGKHKVGPNLHGLIGRKTGQAPGFSYSD      55
Ostrinia furnacalis          MGVPAGNAQNGKKIFVQRCAQCHTVESGGKHKVGPNLHGFFGRKTGQASGSAYSD      55
Papilio machaon              MGVPAGNAENGKKIFVQRCAQCHTVEAGGKHKVGPNLHGFFGRKTGQAPGFTYSE      55
Papilio polytes              MGIPAGNAENGKKIFVQRCAQCHTVEAGGKHKVGPNLHGFFGRKTGQAPGFTYSE      55
Papilio xuthus               MGIPAGNAENGKKIFVQRCAQCHTVEAGGKHKVGPNLHGFFGRKTGQAPGFTYSE      55
Pieris rapae                 MGVPAGNSENGKKIFVQRCAQCHTVEAGGKHKVGPNLHGFYGRKTGQAPGFSYSD      55
Plutella xylostella          MGIPAGNAENGKKIFVQRCAQCHTVEAGGKHKVGPNLNGFFGRKTGQAAGFSYSD      55
Spodoptera litura            MGVPAGNAENGKKIFVQRCAQCHTVEAGGKHKVGPNLHGFWGRKTGQAPGFSYSD      55
Vanessa tameamea             MGVPAGNAENGKKIFVQRCAQCHTVEAGGKHKVGPNLHGFFGRKTGQAAGFSYSE      55
Zeugodacus cucurbitae        MGVPAGDVEKGKKLFVQRCAQCHTVEAGGKHKVGPNLHGLIGRKTGQAAGFSYTD      55
Consensus                    mg pag    gkk fvqrcaqchtve ggkhkvgpnl g  grktgqa g  y

Bombyx mori                  ANKAKGITWNDDTLFEYLENPKKYIPGTKMVFAGLKKANERADLIAYLKSATK      108
Amyelois transitella         ANKAKGISWNEDTLFEYLENPKKYIPGTKMVFAGLKKANERADLIAYLKEATK      108
Bactrocera dorsalis          ANKAKGITWNEDTLFEYLENPKKYIPGTKMVFAGLKKPNERGDLIAYLKSATK      108
Danaus plexippus plexippus   ANKAKGITWGDDTLFEYLENPKKYIPGTKMVFAGLKKANERADLIAYLKEATK      108
Hyposmocoma kahamanoa        ANKSKGITWGDDTLFEYLENPKKYIPGTKMVFAGLKKANERADLIAYLKESTK      108
Ostrinia furnacalis          ANKAKGITWNEDTLFEYLENPKKYIPGTKMVFAGLKKPNERADLIAYLTDATK      108
Papilio machaon              ANKAKGITWGEDTLFEYLENPKKYIPGTKMVFAGLKKANERADLIAYLKEASK      108
Papilio polytes              ANKAKGIIWGDDTLFEYLENPKKYIPGTKMVFAGLKKANERADLIAYLKEASK      108
Papilio xuthus               ANKAKGITWGEDTLFEYLENPKKYIPGTKMVFAGLKKANERADLIAYLKEASK      108
Pieris rapae                 ANKAKGITWSDDTLFEYLENPKKYIPGTKMVFAGLKKANERADLIAYLKDATK      108
Plutella xylostella          ANKSKGISWNEDTLFEYLENPKKYIPGTKMVFAGLKKANERADLIAYLKVATK      108
Spodoptera litura            ANKSKGISWGEDTLFEYLENPKKYIPGTKMVFAGLKKPNERADLIAYLKEATK      108
Vanessa tameamea             ANKAKGITWGDDTLFEYLENPKKYIPGTKMVFAGLKKANERADLIAYLKQATK      108
Zeugodacus cucurbitae        ANKAKGITWNEDTLFEYLENPKKYIPGTKMIFAGLKKPNERADLIAYLKSATK      108
Consensus                    ank kgi w  dtlfeylenpkkyipgtkm faglkk ner dliayl    k
```

Figure S2. Multiple sequence alignment of Bmcytc amino acids with its homologous proteins of other species. The deduced amino acid sequence of Bmcytc and its homologs in other species were retrieved from the NCBI database. Identical amino acids are highlighted in dark blue, and the positive amino acids are highlighted in pink and aqua.

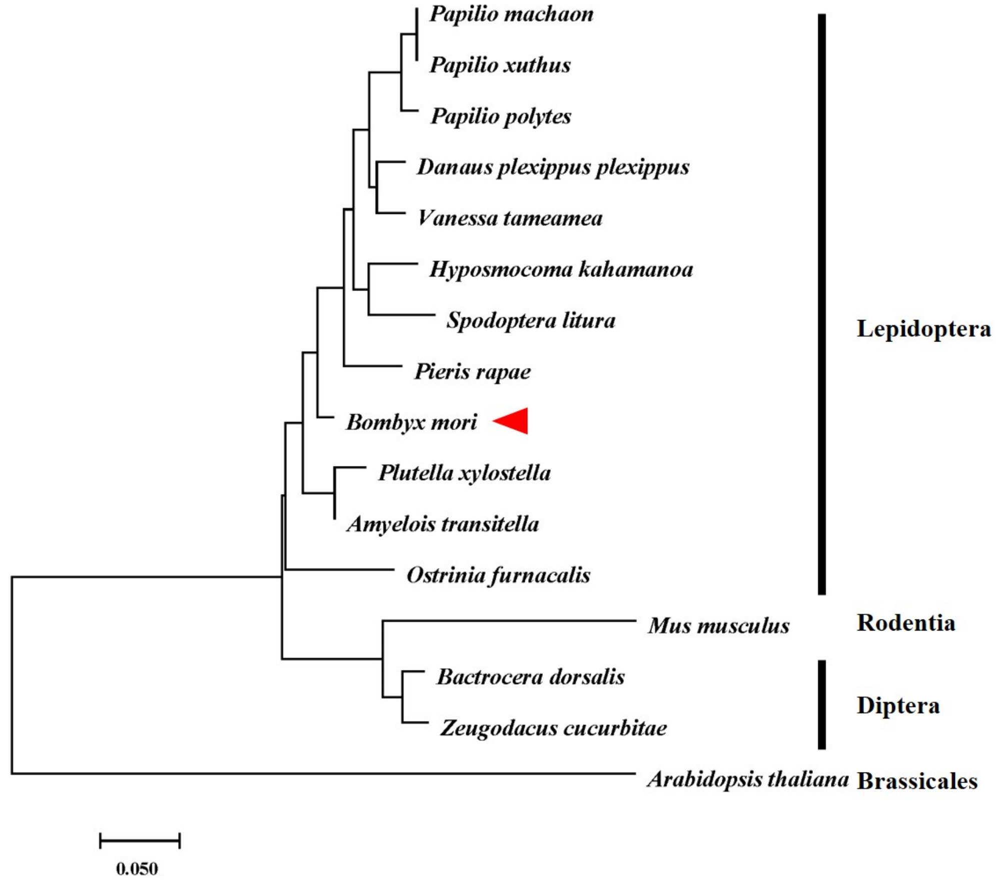

Figure S3. Phylogenetic analysis of *Bmcytc* using the maximum-likelihood method. The tree was constructed with pairwise deletion of gaps in MEGA 7. The percentages on the branches indicate bootstrap values from 1000 replicates. The tree is drawn to scale, with branch lengths in the same units as those of the evolutionary distances used to infer the phylogenetic tree. The evolutionary distances were computed using the *p*-distance method. The analysis involved 16 amino acid sequences. All positions containing gaps and missing data were eliminated. Taxonomic names are shown on the right of the branches.

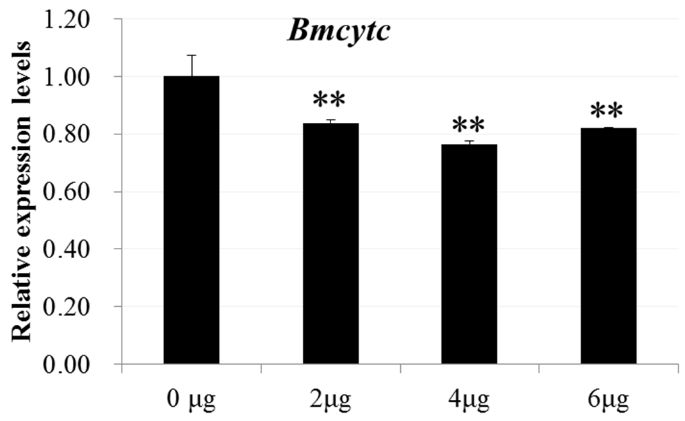

Figure S4. Expression level analysis of *Bmcytc* at different concentrations of siRNA. The data were normalized using *BmGAPDH* and are represented as the mean±standard error of the mean, from three independent experiments. Relative expression levels were calculated using the $2^{-\Delta\Delta Ct}$ method. Statistical analysis was performed using GraphPad Prism 5 software.
